# Real-world sensor dataset for city inbound-outbound critical intersection analysis

**DOI:** 10.1038/s41597-022-01448-6

**Published:** 2022-06-21

**Authors:** Ei Ei Mon, Hideya Ochiai, Patrachart Komolkiti, Chaodit Aswakul

**Affiliations:** 1 Wireless Network and Future Internet Research Unit, Department of Electrical Engineering, Faculty of Engineering, Chulalongkorn University, Bangkok, 10330 Thailand; 2 Information and Communication Engineering, Graduate School of Information Science and Technology, The University of Tokyo, Tokyo, 113-8656 Japan; 3 Learning Innovation Center, Chulalongkorn University, Bangkok, 10330 Thailand

**Keywords:** Electrical and electronic engineering, Civil engineering

## Abstract

This paper reports the release of dataset that describes the critical city inbound-outbound intersection in the central business district’s Sathorn area of Bangkok, Thailand. The available traffic volume and occupancy are captured by sensors located on the Sathorn area’s links. Induction loop coil sensors and thermal and CCTV cameras have been installed at the approaching links of the critical Sathorn-Surasak intersection. Traffic volume data have been collected from the CCTV cameras every 5 s from 2016 to 2019, and occupancy and volume data have been collected from the loop coil sensors every 5 s from May to September 2016 during a social experiment that was part of Sathorn Model project. Occupancy and volume data have also been collected from the thermal cameras every 5 s from May to June 2016. The dataset provides temporal and spatial coverage of Sathorn Road’s primary urban areas, including weekdays, weekends, and public holidays. This dataset can be resources for research on traffic state estimation, traffic light control optimization, and the analysis of critical intersections.

## Abstract (summary)

**Table:** 

Measurement(s)	traffic data
Technology Type(s)	cctv camera • thermal camera • loop coil • taxi

## Background & Summary

The transportation sector faces enormous challenges, primarily due to the continuous increase in vehicular traffic worldwide, particularly in urban areas. It is thus necessary to capture traffic information such as vehicles, individual vehicle speed, and movement direction. To obtain such information, government organizations have installed roadside sensing infrastructure such as cameras and sensors to collect traffic and environmental condition data.

Roadside sensors, such as induction loop detectors, magnetic sensors, and closed-circuit television (CCTV) cameras, are widely used in the planning and development of urban cities. Additionally, the information obtained from these sensors provides commuters with trip guidance. Induction loop detectors can detect the number, occupancy, and movement of vehicles. Video cameras can also detect vehicles passing through lanes. These sensors can be used to classify vehicles according to vehicle length and provide detailed information such as speed, occupancy, and flow rate. In addition, mobile sensors such as cellular phones and portable hand-held global positioning system (GPS)-embedded devices are alternatives to fixed-location sensors (e.g., loop detectors). Using vehicles equipped with GPS sensors as probes is an interesting approach for traffic data collection to help increase geographic coverage. With the increasing use of traffic data collection sensors, the data obtained from such sensors have become more reliable for use in traffic monitoring.

Traffic sensor data provide detailed traffic information such as traffic volume and vehicle speed at the link level and the lane level. Local transportation agencies require traffic volume information to mitigate traffic congestion, e.g., to adjust traffic signal timing or to change the allowable vehicle movement direction to accommodate time-varying demands on a particular road. Pollution monitoring systems have also used traffic volume as input data to compute vehicle emissions and total gas consumption^1^.

Openness in data sharing has increased enormously worldwide in all application domains. For transportation engineering, it is essential to consider how to collect historical traffic information from sensors to forecast future traffic conditions^2^. However, the full advantage of open data has yet to be explored in urban science. The lack of a publicly available dataset with sufficient duration hinders transportation research. Analyzing historical traffic data can yield valuable insights such as travel time predictions and traffic bottleneck analysis^3^ and real-time movement-based traffic volume prediction^4^. Today, transportation data are publicly available in many parts of the world^5^. Publicly available transportation datasets are classified as real-world sensor datasets, annual reports, real-world GPS datasets, application program interface (API)-accessible datasets, and simulated datasets.

### Real-world sensor dataset

Traffic flow data are recorded as time-series data. Real-time traffic data^6^ from Åarhus are recorded every five minutes to monitor city mobility. NYC open data^7^ has released the street’s real-time traffic speed data primarily on major arterials and highways with a sampling frequency of five minutes. The freeway performance measurement system (PeMS)^8^ provides real-time traffic data from automatic sensors installed on most California freeways. Its real-time and historical data include real-time freeway performance metrics such as travel times and freeway bottlenecks. Uber has launched Uber Movement^9^, which openly provides traffic data to retrieve information about travel time and vehicle travel speeds in different parts of the world. The digital roadway interactive visualization and evaluation network (DRIVE Net)^10^ provides loop detector data, incident data, probe vehicle data, and weather data for the Washington State Department of Transportation (WSDOT) regions; it also provides functions to calculate the level of service (LOS), estimate traffic-related emissions, predict crashes and analyze and visualize different transportation data.

### Annual report

Worldwide, public administrations and government agencies are publishing open data, which have been critical to the smart city movement for many years. Municipalities provide access to helpful datasets using data integration and visualization tools that create graphic charts based on the dataset’s values. The dataset^11^ is an aggregated time-series dataset of annual road traffic fatalities in Great Britain (GB). GB road traffic count data are available for each intersection link on the major road network. In Bangkok, Thailand, the Bangkok Metropolitan Administration has provided the annual intersection traffic flow survey^12^ for all major intersections in the city with a data resolution of fifteen minutes. However, the surveys were conducted manually without automatic sensor deployment.

### Real-world GPS dataset

For traffic monitoring and urban planning, taxi trajectories reflect real spatial-temporal traffic conditions, such as traffic conditions of each road within the city area at any time interval. These traffic conditions are advantageous for traffic volume estimation^13,14^. The dataset^15^ reports the mobility traces of taxis in Rome, Italy, using 316 taxies equipped with a GPS receiver and collected over 30 days to retrieve the information of vehicles’ geolocations. A thirteen-year (2009–2021) NYC Taxi and Limousine Commission has published taxi trip records^16^ in New York City with taxi pick-up and drop-off locations. Tsinghua University has provided a taxi trajectory dataset^17^ of Beijing collected in May 2009 with 129 million data samples. The GPS trajectory dataset^18^ provides urban context information, including the accuracy level, bearing, speed, and label trajectories using two kinds of traffic modes (car and motorcycle). This dataset covers more than 1 million km of roads.

### API accessible dataset

Transport navigation systems constitute a new area of applied recommendation systems. Navigation services such as Google Maps^19^ provide travel time estimation and calculation of routing functions. Results from navigation services are based on several real-world traffic sources, including statistics produced by transportation agencies, floating car data collected by in-vehicle systems, and crowdsourced data collected by GPS-enabled smartphones. The resulting information is provided to commuters via the API. Traffic data providers such as TomTom^20^, HERE^21^, INRIX^22^, and Google Maps^19^ typically provide aggregated historical and real-time traffic information. NYC open data^7^ also provide real-time traffic speed information via the API. The dataset^23^ has street-level traces of traffic flow for 13 cities in Romania with a sampling interval of 15 min, which is consistent with the HERE API^21^.

### Simulated dataset

The realistic synthetic dataset^24^ for large-scale urban vehicular mobility in the city of Köln, Germany, covers an area of 400 *km*^2^ over 24 hours by considering a realistic road topology, individual driver behaviors, and large-scale traffic flows. Traffic simulation is a widely used method that is applied in traffic modelling, planning, traffic networks, and systems development. For example, simulation of urban mobility (SUMO)^25^ has been broadly used to investigate several research topics, such as vehicle-to-vehicle (V2V) communication systems, vehicle interactions, traffic optimization, and route choice. For example, the Bologna Ringway dataset^26^, which uses SUMO, describes the traffic flow during the morning peak hour of more than 22 000 vehicles in an area of 25 *km*^2^ covering the capital of Bologna, Italy. Chula-SSS^27^ supports two calibrated datasets (morning and evening) using SUMO^25^ for the Sathorn road network area in Bangkok, Thailand. The simulated Chula-SSS covers 2375 intersection nodes, 4517 edges and ten signalized intersections.

### Proposed dataset for the critical inbound-outbound intersection

With advanced data collection technologies, most studies have attempted to evaluate the signalized intersection performance using various data sources^28^. In Bangkok, Thailand, the Sathorn Model has been used to find a sustainable solution for traffic congestion problems in the Sathorn area of Bangkok. Activities are managed by the Faculty of Engineering, Chulalongkorn University, and Sathorn Road serves as the gateway linking the residential area on the western side of the Chao Phraya River and the river-eastern side’s business area, as shown in Fig. 2. As a result, up to 150,000 vehicles drive every weekday on Sathorn Road. Sathorn district has been chosen as an experimental area for two primary reasons. First, it is a critical central business district (CBD) that experiences oversaturated traffic conditions and the slowest travel speeds in Bangkok^29^. Second, additional development is nearly impossible due to existing infrastructure. Therefore, it is crucial to create better mobility via traffic flow management^30^. For those reasons, we planned to measure traffic flow using different sensors for a critical inbound-outbound intersection, particularly the Sathorn-Surasak intersection in the Sathorn area.

**Fig. 1:**
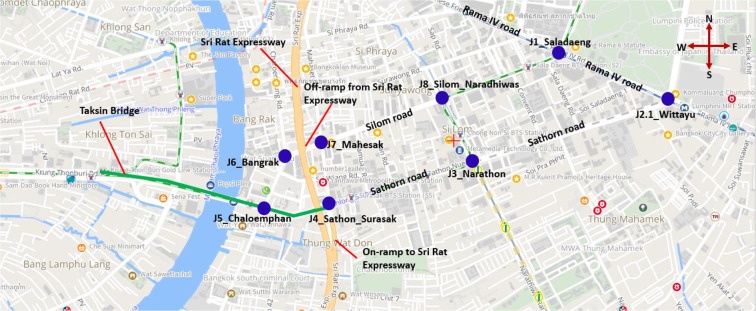
Intersection locations in network-wide area comprehensive manual traffic data collection.(Note that all road types are left-handed-traffic since Thailand is a left-handed-traffic and right-handed-driving country^27^) [Source of base map: http://map.longdo.com/].

Road coverage measures describe the density aspect of the elements of a network, as intersections and links. Intersection density attributes road network coverage in an area. Coverage measures help determine compactness and development. Before measuring the traffic data using real-world sensors in the critical inbound-outbound Sathorn-Surasak intersection, we have tried to collect the data manually in the extensive coverage area on the same sample day, 31 July 2014, for morning and evening rush hours from 6 AM to 9:30 AM and 2 PM to 8:30 PM. We have considered this sample dataset a significant dataset to understand the traffic conditions of the whole area. Before such attempts, due to lack of resources, traffic data collection must also be done manually and within a small area (e.g., within a single intersection area) each day. Getting the whole dynamics from all the related intersections had been impractically challenging. This whole-area traffic data collection done all at the same day and time periods was thus considered a significant contribution to quantifying and understanding the whole area situation. The data collection area is shown in Fig. 1. By analyzing these whole-area traffic conditions, we have obtained clear evidence suggesting that the Sathorn-Surasak intersection was the most critical. The gateway controls inbound and outbound traffic between the Bangkok business district east of Taksin bridge and the residential area in the west. And that was the main reason why all subsequent automatic traffic data collection via sensors focused on all traffic approaching directions around that critical intersection.

The Sathorn-Surasak intersection has both inbound and outbound traffic flows. There are three types of sensors installed on the approaching links of the Sathorn-Surasak intersection in the Sathorn area: CCTV cameras, thermal cameras, and induction loop coil sensors. Traffic volume data have been collected every 5 s from CCTV sensors for 37 months from September 2016 to September 2019. Traffic volume and occupancy have also been monitored every 5 s by loop coil sensors for 110 days from May 2016 to September 2016. Additionally, traffic volume and occupancy have been monitored every 5 s by thermal cameras for 26 days from May 30, 2016, to June 24, 2016. These measures have been first tested over the whole Sathorn road network within the three-week social experiment period from May 30 to June 24, 2016, to evaluate their effectiveness and practicality. Once we have identified effective measures, we have continued to measure and collect the data for the long run^27^. The dataset has temporal and spatial coverage of Sathorn Road’s primary urban areas, including on weekdays, weekends, and public and national holidays. This research has never been achieved before in Bangkok at this scale of data resolution. Although other datasets have already been provided for urban areas in other cities, road infrastructures and road types are not the same for every city. A unique aspect of this dataset is its description of a critical inbound-outbound intersection in a CBD. The richness of the dataset for the inbound-outbound traffic across this Sathorn-Surasak intersection is helpful for future research focusing on a critical intersection’s high-resolution characteristics. The dataset provided in this study is also expected to be beneficial to other researchers who want to address other large cities, which share similarities in critical intersections that serve as gateways between the city’s work and residential zones.

The dataset developed in this study provides an opportunity for the research community to make a significant impact by performing deeper investigations into traffic signal optimization and queue length estimation. We believe that the release of this dataset will benefit the community because it will provide a foundation for benchmarks for real-time traffic prediction. While other traffic datasets represent urban city areas, this dataset serves as a city scenario in a populous city in Southeast Asia. Additionally, the distinct mobility features and oversaturated congestion pose a challenge to local practitioners and the traffic engineering research community globally. However, evidence suggests traffic police have expertise with explicit, tacit knowledge.

## Methods

### Preliminary manual collected data

For manual data collection, we have first selected eight intersections in the extensive coverage area to measure the density aspect of the elements of a network, as intersections and links, including the Sathorn-Surasak intersection relevant to the critical inbound-outbound intersection of the central business district. The selected intersections and their locations are shown in Fig. 1. We have collected data with team members for each responsible location in the whole coverage network area on the same sample day, Thursday, 31 July 2014, for morning and evening rush hours from 6 AM to 9:30 AM and 2 PM to 8:30 PM. Each member has videoed the traffic conditions with a mobile phone to record video for the whole observation period. The raw recorded videos have been replayed, and our team members manually have extracted wanted traffic data of interest. The data extraction process has been executed by replaying the recorded videos to ensure accuracy that reflects the traffic conditions on that traffic census day. Three traffic data types: volume, signal, and queue length, have been collected manually. Traffic volume data serves multiple needs for transportation experts and is vital to establishing the baseline conditions for roadways and intersections. Traditional traffic data collection techniques include manual turning movement counts to quantify vehicles on highways or at an intersection. We also have collected traffic counts turning ratio volume data for each intersection depending on the need. We have utilized this collected whole-area traffic dataset on the traffic census day to understand the traffic congestion state that would potentially link to the critical intersection analysis in this area.

### CCTV camera

**Fig. 2:**
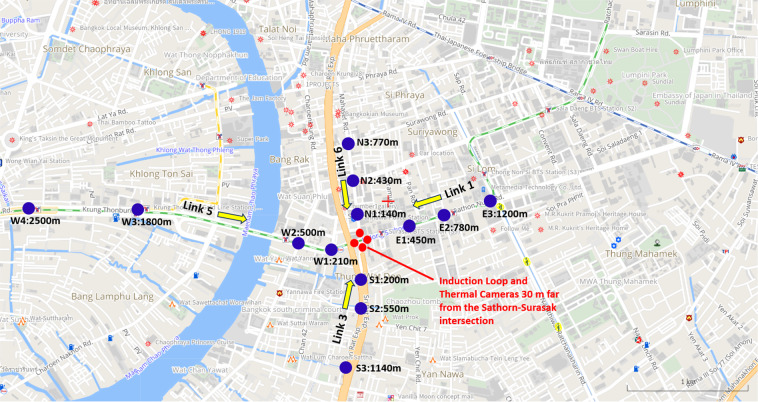
Sensor locations (CCTV cameras with blue color, thermal and loop coil sensors with red color) in the Sathorn model project. (Note that all road types are left-handed-traffic since Thailand is a left-handed-traffic and right-handed-driving country^27^) [Source of base map: http://map.longdo.com/].

Video cameras provide data and are generally preferable to alternative data sources. Video cameras do not require direct intervention to install magnetic loops on the streets or various other expensive infrastructure types. Video tracking cameras are used to measure the traffic volume, flow and velocity of all vehicles. There are a total of 13 sensors (three sensors on Surasak road (Link 6), three sensors on Charoen Rat Road (Link 3), three sensors on South Sathorn Road (Link 1), and four sensors on North Sathorn Road (Link 5)). Sensors *W*1 and *W*2 are on the Taksin bridge of Link 5. The detailed configuration of the physical sensor placement is shown in Fig. 2. These sensors are on all approaching links of the Sathorn-Surasak intersection to capture traffic volume. Note that all road types are left-handed-traffic since Thailand is a left-handed-traffic and right-handed-driving country^27^. For data collection using CCTV and thermal-CCTV cameras, we have used a cost-effective TrafiCam x-stream2^31^ sensor to detect moving and stationary vehicles at the intersection and approaching links of the Sathorn-Surasak intersection, as shown in Fig. 2.

### Loop coil sensor

**Fig. 3:**
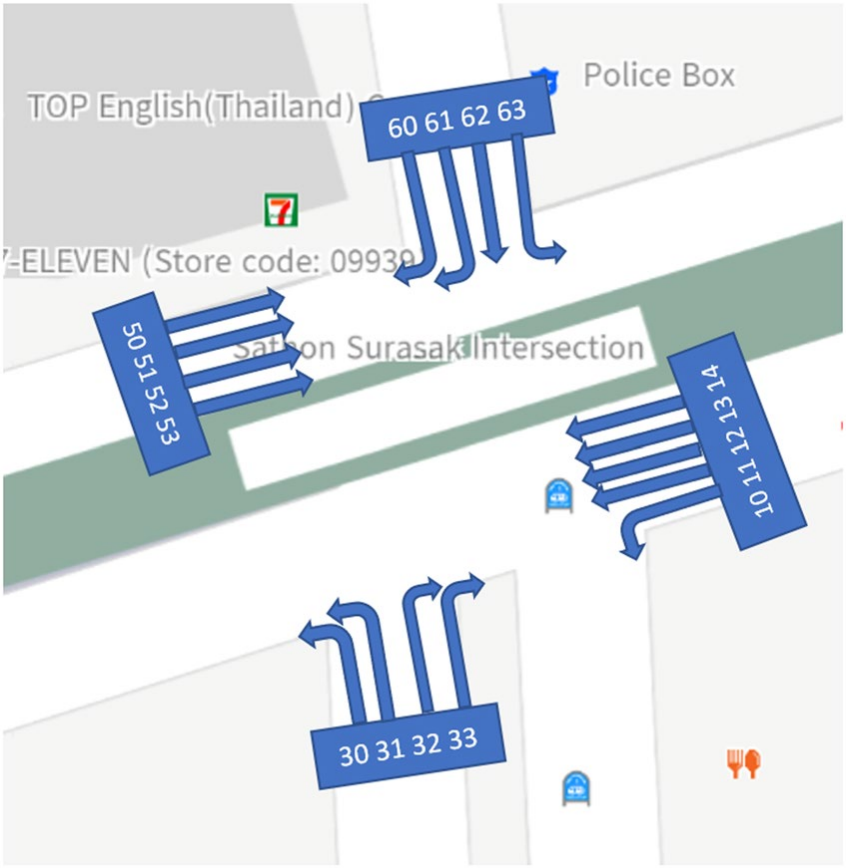
Loop coil sensors and thermal camera locations. Sensors on specific links are 30 m from the Sathorn-Surasak intersection. [Source of base map: http://map.longdo.com/].

Induction loop detectors count the vehicles and detect the movement and occupancy of vehicles passing or arriving at a certain point through the conducting loop installed in the pavement. These loop detectors have been widely used due to their simple electronic circuitry, long life span, low cost, and design to detect large vehicles such as cars, buses, and trucks. However, they cannot recognize small vehicles such as motorcycles and bicycles with good accuracy^32^. Induction loop detectors are primarily installed in different locations near traffic lights. There are 17 loop coil sensors at the Sathorn-Surasak intersection to capture traffic volume and occupancy for all traffic movement directions, as shown in Fig. 3.

### Thermal camera

There are four thermal cameras with 17 virtual sensor points for all approaching lanes at the Sathorn-Surasak intersection to capture traffic volume and occupancy. The locations of the thermal cameras are the same as those of the loop coil sensors, as shown in Fig. 3.

### Signal control sensor

**Fig. 4:**
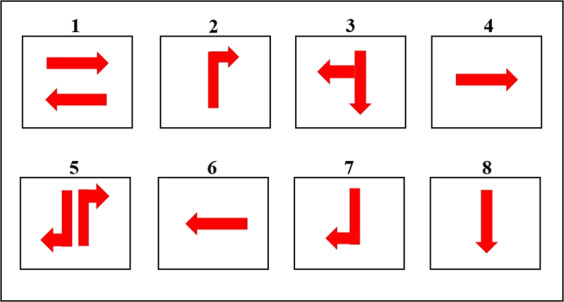
Traffic light phases used in the Sathorn-Surasak intersection.

Traffic signal timing in real or near-real time requires the continuous processing of massive volumes of traffic movement information, including traffic flow and speed. At the Sathorn-Surasak intersection, there are eight main traffic light phases, as shown in Fig. 4. Phase 1 is for both traffic directions on North and South Sathorn Roads. Traffic flows from west to east and from east to west. Phase 3 is for only vehicles on Surasak Road going straight south or turning right. Concurrently, vehicles in the leftmost lane of Surasak Road can also turn left onto North Sathorn Road. Phase 5 is only for the two flows that are enabled: the first is the flow from Surasak Road turning right to Taksin bridge, and the second is the flow from Charoen Rat Road turning right onto North Sathorn Road. Phase 4 is only for the flow on North Sathorn from west to east. Phase 6 is only for the flow on South Sathorn road from east to west. Phases 2 and 7 are partly enabled by Phase 5. Additionally, Phase 8 is partly enabled by Phase 3. Note that Phases 2, 4, 6, 7 and 8 are intended to be used only when queue spill-back events occur so specific movement directions are disabled.

### Taxi fleet

**Fig. 5:**
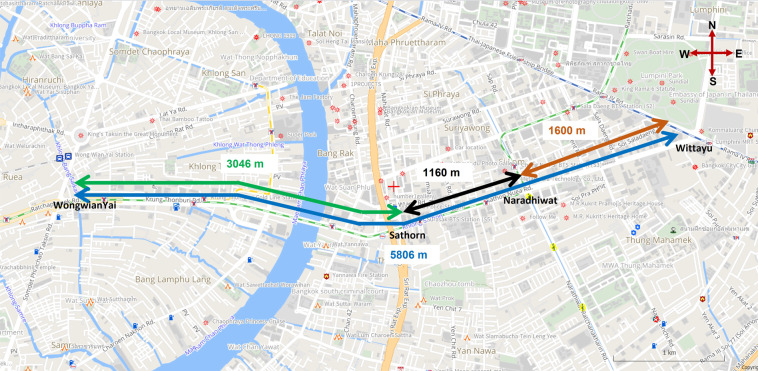
Sathorn area. (Note that all road types are left-handed-traffic since Thailand is a left-handed-traffic and right-handed-driving country^27^) [Source of base map: http://map.longdo.com/].

**Fig. 6:**
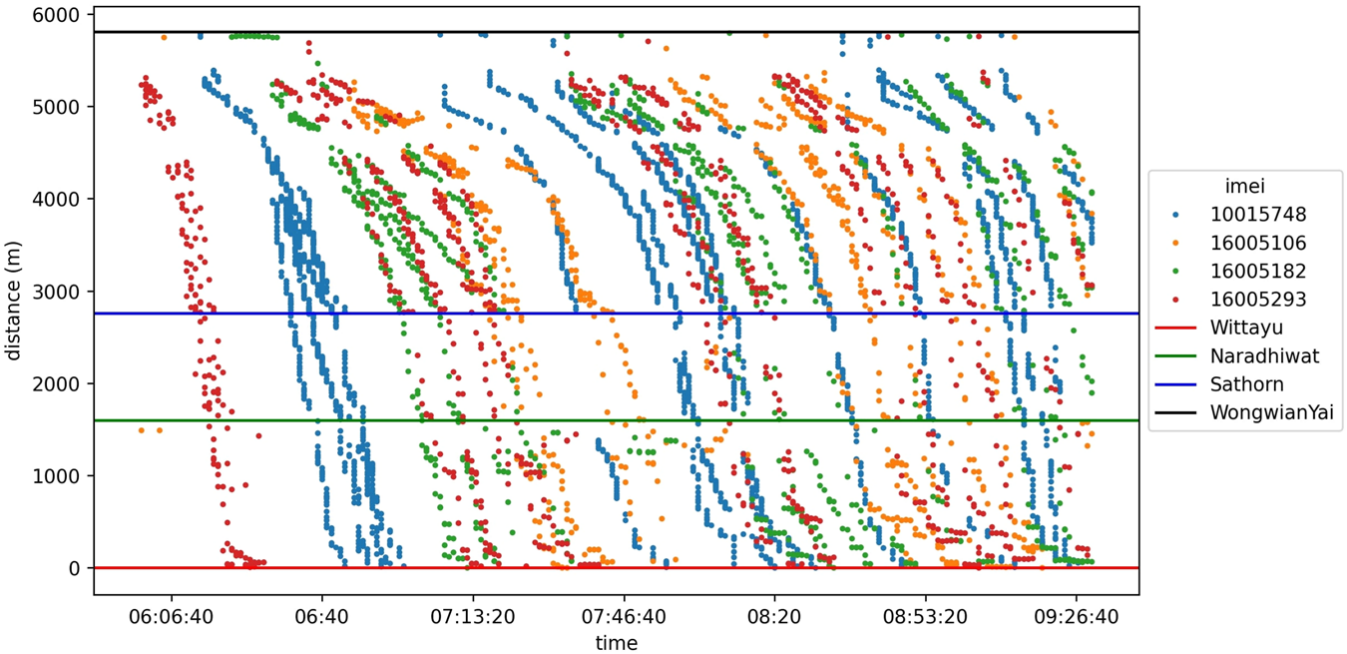
Taxi trajectory data in the Sathorn Road area on all six evaluation days during the social experiment.

Speed is also a helpful parameter to represent traffic state conditions. Vehicles equipped with a global positioning system (GPS) are an enriched data source for traffic research fields. GPS-equipped vehicles are widely used to analyze traffic phenomena and can collect and output mobility data periodically, including longitude, latitude, speed, vehicle headings, and timestamps. GPS vehicle data are commonly recorded as a trajectory; thus, GPS-equipped vehicles are used to collect traffic speed data for the Sathorn area. The primary origin-destination is shown in Fig. 5, and Fig. 6 shows the morning taxi trajectory data in the Sathorn Road area.

## Data Records

Firstly, the traffic data from the relevant intersections are collected manually on the same sample day, 31 July 2014, for morning and evening rush hours from 6 AM to 9:30 AM and 2 PM to 8:30 PM. After getting clear evidence from manually collected data, we have started collecting the data from real-world sensors. Traffic volume data have been collected from CCTV cameras every 5 s from 2016 to 2019 for 37 months. The occupancy and volume monitored loop coil sensors have been recorded every 5 s from May 2016 to September 2016 for 110 days during the social experiment of the Sathorn Model project. The occupancy and volume monitored by thermal cameras have been recorded every 5 s from May 2016 to June 2016 for 26 days for data validation purposes of other corresponding sensors. Signal control data have been collected from November 2014 to September 2016 for 22 months. Taxi speed data have been collected for only six days to study the before-and-after effects of the trial measures of traffic flow management being implemented during the social experiment of the Sathorn Model project. The available dates of each data type are shown in Table 1. All data have been uploaded and can be accessed from figshare^33^.

**Table 1:** Available dates and intended usages of each data type.

Sensor	Data	Available Dates	Intended Usages
Manual collection	Volume Signal data Queue length	31-Jul-2014	Data collection for understanding the whole area of traffic conditions all at once before we started collecting the real-world sensors data of critical intersection. The indicators undertaken to measure the large coverage area of the road network can be applied to identify the inbound-outbound intersection of the city’s business district area.
CCTV camera	Volume	13-Sep-2016 to 23-Sep-2019	Data collection expansion resulted from a thermal CCTV-based sensor feasibility study during a social experiment that was part of the Sathorn Model project. The software used for video signal processing of CCTV and thermal CCTV sensors is the same. The normal grade of CCTV (without thermal detection) has been selected to determine occupancy values but was disabled for cost-effectiveness during this scaled-up installation.
Loop Coil	Volume Occupancy	27-May-2016 to 13-Sep-2016	Data collection during the social experiment of the Sathorn Model project to validate the thermal CCTV-based sensors.
Thermal CCTV camera	Volume Occupancy	30-May-2016 to 24-June-2016	Data collection for the feasibility study of CCTV-based sensor type in real deployment conditions during the social experiment of Sathorn Model project.
Signal Control	Signal data	9-Nov-2014 to 14-Sep-2016	Data collection for short-term and long-run behaviors of how traffic police operate traffic signal light controls during rush hour periods or switch to automatic pre-timed traffic signal light settings during other periods.
Taxi	Speed	31-May-2016, 2-June-2016, 9-June-2016, 14-June-2016, 16-June-2016, 23-June-2016	Data collection to study before-and-after effects of the traffic flow management trial measures being implemented during the social experiment of the Sathorn Model project.

### Data from manual collection

Data are segmented for each intersection as shown in Table 2. Each.csv file consists of data samples for the morning and evening rush hours from 6 AM to 9:30 AM and 2 PM to 8:30 PM. The structure of traffic volume and queue length is shown in Table 3, and the structure of traffic signal is shown in Table 4.

**Table 2:** Data files of manual collected traffic data (volume, signal data, queue length) for each intersection.

Name	Description
[intersection name]_[data type].csv	Data files. The value of ‘[intersection name]’ part of the filename is the intersection name that have been considered as relevant intersection of critical inbound-outbound intersection. The value of the ‘[data type]’ is the manual collected data type such as volume, signal data and queue length.

**Table 3:** Descriptions of manually collected data (volume and queue length).

Attributes	Description
time	Collected time interval (24 hours format).
from north	Number of vehicles collected according to the turn ratio (L,S,R,U) and queue length in each traffic movement direction of intersection.
from east	
from west	
from south	

Remark: There is no U-turn in some traffic movement direction.

**Table 4:** Description of manually collected traffic signal light data attributes.

Attributes	Description
Time	Time in which the instance is collected (HH/MM/SS).
Phase	The phase in which the instance is collected.
Duration	The duration of time in which the traffic light phase is operated.

Remark: For example, three data attributes with [6:00:13, 4, 24] means duration of phase 4 is 24 s and this phase started from 6:00:13 AM.

### Data from CCTV camera

Data are segmented into days for each link. Each folder consists of files separated by each day, as shown in Table 5. For example, Link 1 has three sensors (E1, E2 and E3), and each.csv file consists of data samples with the structure shown in Table 6. The downloadable datasets for each link between 2016 and 2019 are shown in Table 7. Each file includes data from all sensors on each link, as shown in Fig. 2. Table 8 shows the missing days of data from CCTV cameras for each link.

**Table 5:** Data files of traffic volume from CCTV cameras on each link.

Name	Description
[date]_volume_[link].csv	Volume files. The value of ‘[date]’ part of the filename is the real date when the volume data are collected and saved in the file, and the value of the ‘[link]’ is l1, l3, l5 and l6, corresponding to Link 1, Link 3, Link 5 and Link 6, respectively. For instance, 20161001_volume_l3.csv refers to the volume data file for Link 3 on October 1, 2016.

**Table 6:** Descriptions of data attributes collected from CCTV cameras.

Attributes	Description
Time	Starting time of the 5 second interval in which the instance is collected (24 hours format).
[N/E/W/S] 1	Number of vehicles collected from the first sensor on the link approaching the intersection from corresponding directions [N: north, E: east, W: west, S: south].
[N/E/W/S] 2	Number of vehicles collected from the second sensor on the link approaching the intersection from corresponding directions [N: north, E: east, W: west, S: south].
[N/E/W/S] 3	Number of vehicles collected from the third sensor on the link approaching the intersection from corresponding directions [N: north, E: east, W: west, S: south].
[N/E/W/S] 4	Number of vehicles collected from the fourth sensor on the link approaching the intersection from corresponding directions [N: north, E: east, W: west, S: south](the fourth sensor exists only on Link 5).

**Table 7:** Data availability of traffic volume from CCTV cameras on each link.

	2016	2017	2018	2019
Link 1	20160913_volume_l1.csv to 20161231_volume_l1.csv	20170101_volume_l1.csv to 20171231_volume_l1.csv	20180101_volume_l1.csv to 20181231_volume_l1.csv	20190101_volume_l1.csv to 20190923_volume_l1.csv
Link 3	20160913_volume_l3.csv to 20161231_volume_l3.csv	20170101_volume_l3.csv to 20171231_volume_l3.csv	20180101_volume_l3.csv to 20181231_volume_l3.csv	20190101_volume_l3.csv to 20190923_volume_l3.csv
Link 5	20160913_volume_l5.csv to 20161231_volume_l5.csv	20170101_volume_l5.csv to 20171231_volume_l5.csv	20180101_volume_l5.csv to 20181231_volume_l5.csv	20190101_volume_l5.csv to 20190923_volume_l5.csv
Link 6	20160913_volume_l6.csv to 20161231_volume_l6.csv	20170101_volume_l6.csv to 20171231_volume_l6.csv	20180101_volume_l6.csv to 20181231_volume_l6.csv	20190101_volume_l6.csv to 20190923_volume_l6.csv

**Table 8:** Missing dates of data from all CCTV cameras on each link.

	2017	2018	2019
Link 1	20170122, 20170127, 20170128, 20170427, 20170817, 20170818	20180705, 20180706, 20180707, 20180708, 20180709, 20180710, 20180711, 20180712, 20180713, 20180714, 20180715, 20181212	20190504, 20190507
Link 3	20170122, 20170127, 20170128, 20170817, 20170818	20180705, 20180706, 20180707, 20180708, 20180709, 20180710, 20180711, 20180712, 20180713, 20180714, 20180715	—
Link 5	20170122, 20170127, 20170128, 20170817, 20170818	20180705, 20180706, 20180707, 20180708, 20180709, 20180710, 20180711, 20180712, 20180713, 20180714, 20180715	—
Link 6	20170122, 20170127, 20170128, 20170817, 20170818	20180705, 20180706, 20180707, 20180708, 20180709, 20180710, 20180711, 20180712, 20180713, 20180714, 20180715	—

### Data from the loop coil sensor and thermal CCTV camera

Data are segmented into days for each link. Each folder consists of files that are separated by each day for each sensor. For example, Link 1 has five sensors (10, 11, 12, 13 and 14), as shown in Table 9. The data format and description of the loop coil sensors with the available data from May 27, 2016, to September 13, 2016, are shown in Tables 9 and 10, respectively. As shown in Fig. 3, loop coil sensors and thermal cameras are at the same location and collect the same data type. Therefore, thermal cameras also have the same data format and description as the available data from May 30, 2016, to June 24, 2016, as shown in Tables 9 and 10, respectively.

**Table 9:** Data availability of traffic volume from loop coil sensors and thermal CCTV cameras for each link.

	Link 1	Link 3	Link 5	Link 6
May 2016	20160530_[sensor].csv-20160531_[sensor].csv The value of [sensor] part of the filename is sensor of Link 1 (10, 11, 12, 13, 14).	20160530_[sensor].csv-20160531_[sensor].csv The value of [sensor] part of the filename is sensor of Link 3 (30, 31, 32, 33).	20160530_[sensor].csv-20160531_[sensor].csv The value of [sensor] part of the filename is sensor of Link 5 (50, 51, 52, 53).	20160530_[sensor].csv-20160531_[sensor].csv The value of [sensor] part of the filename is sensor of Link 6 (60, 61, 62, 63).
June 2016	20160601_[sensor].csv-20160624_[sensor].csv The value of [sensor] part of the filename is sensor of Link 1 (10, 11, 12, 13, 14).	20160601_[sensor].csv-20160624_[sensor].csv The value of [sensor] part of the filename is sensor of Link 3 (30, 31, 32, 33).	20160601_[sensor].csv-20160624_[sensor].csv The value of [sensor] part of the filename is sensor of Link 5 (50, 51, 52, 53).	20160601_[sensor].csv-20160624_[sensor].csv The value of [sensor] part of the filename is sensor of Link 6 (60, 61, 62, 63).
Remark	20160604, 20160605, 20160611, 20160612, 20160618, 20160619 are missing days for all thermal cameras on all links. The available dates of loop coil sensors start from May 27, 2016, to September 13, 2016.

**Table 10:** Description of data attributes collected from loop coil sensors and thermal CCTV cameras.

Attributes	Description
Date	Date on which the instance is collected (DD/MM/YY).
Time	Starting time of the 5 s interval in which the instance is collected (24 hours format).
Volume	Number of vehicles (in passenger-car units: PCUs) that enter the sensor location in the previous 5 s time interval. For example, at 00:00:20 with the volume of 1 means that during 00:00:15-00:00:20, there is one vehicle passing through the sensor location.
Occupancy (%)	The proportion of time in which vehicles occupy the sensor, whether passing through the sensor or staying still. For example, at 00:00:20 with the occupancy is 26% which means that during 00:00:15-00:00:20, there are approximately 1.3 s during which the sensor can capture vehicles.

### Signal data

Signal data are segmented into days. Each.csv file consists of data samples with the structure shown in Table 11. Each (.csv) file consists of data samples with the structure shown in Table 12. Downloadable data are available from November 19, 2014, to September 14, 2016. There are also 176 missing days in signal data within 22 months from November 19, 2014, to September 14, 2016. For those missing days, we have shown and listed them in the Jupyter notebook.

**Table 11:** Data files of traffic signal light.

Name	Description
[date].csv	Signal files. The value of ‘[date]’ is the date when the signal data are collected and saved in the file.

**Table 12:** Description of traffic signal light data attributes.

Attributes	Description
Time	Time in which the instance is collected (HH/MM/SS).
Mode	The mode in which the traffic light is operated; 1 = Automatic, 2 = Manual.
Phase	The phase in which the instance is collected. For example, four data attributes with [10:25:55, 2, 1, 45] means duration of phase 1 is 45 s with mode 2 at 10:25:55 AM.
Duration	The duration of time in which the traffic light phase is operated. For example, four data attributes with [10:25:55, 2, 1, 45] means the duration of phase 1 is 45 s with mode 2 at 10:25:55 AM.

## Technical Validation

With transportation management, analysis with data refers to processes that extract implicit knowledge and rules from a large amount of traffic data that govern the movement of traffic under different real-world conditions^34^. This section presents data visualization to show the dataset’s quality and technical validity, including daily and weekly pattern plots and weekday and weekend pattern plots. In addition, the missing data plot provides insights into data availability.

### Manual collected traffic data

**Fig. 7:**
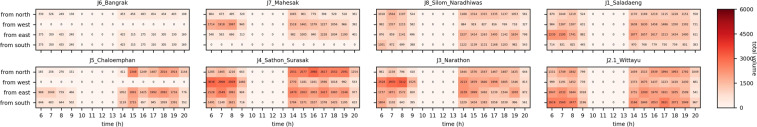
Total volume for each turning movement direction of each intersection in whole network-wide area on manual traffic data collection day.

**Fig. 8:**
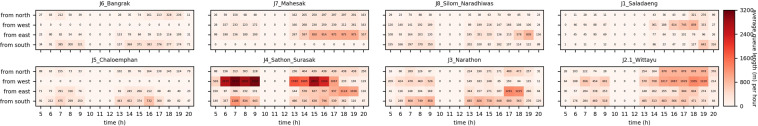
Average queue length per hour for each turning movement direction of each intersection in whole network-wide area on manual traffic data collection day.

Figures 7 and 8 show the manual collected traffic volume and average queue length per hour for each intersection on the same sample day, 31 July 2014, from 6 AM to 9:30 AM for volume and 5 AM to 9:30 AM for queue length, and 2 PM to 8:30 PM. The observed traffic conditions are shown in Figs. 7 and 8 according to the location of each intersection as shown in Fig. 1. In Figs. 7 and 8, the white color with 0 indicates that we have not collected data from 10 AM to 12 PM on this sample day in the manual collection. There is no data from the traffic movement from the west in Bangrak and Chaloemphan intersections.

Figure 7 shows the total traffic volume for each traffic movement direction using turning ratio volume data. The observed traffic conditions in Chaloemphan, Sathon-Surasak, Narathon, and Wittayu intersections along the Sathorn road indicate that vehicles have mainly used Sathorn road to commute to the business district area, as shown in Fig. 7. Apart from the Sathorn road, commuters have used the Sri Rat expressway to come to this business area. As shown in the first row of Fig. 7, Mahesak, Silom-Naradhiwas, and Saladaeng also have traffic demands from Sri Rat expressway and Rama IV road to commute the area.

According to the reported results of average queue length per hour in Fig. 8, the queue length data from the west in the Sathorn-Surasak intersection has indicated the severe traffic condition according to the thickness of the color bar in the heatmap. The reported queue length shows that the Sathorn-Surasak intersection mainly allows commuters to enter the residential area and exit the work zone and vice visa.

**Fig. 9:**
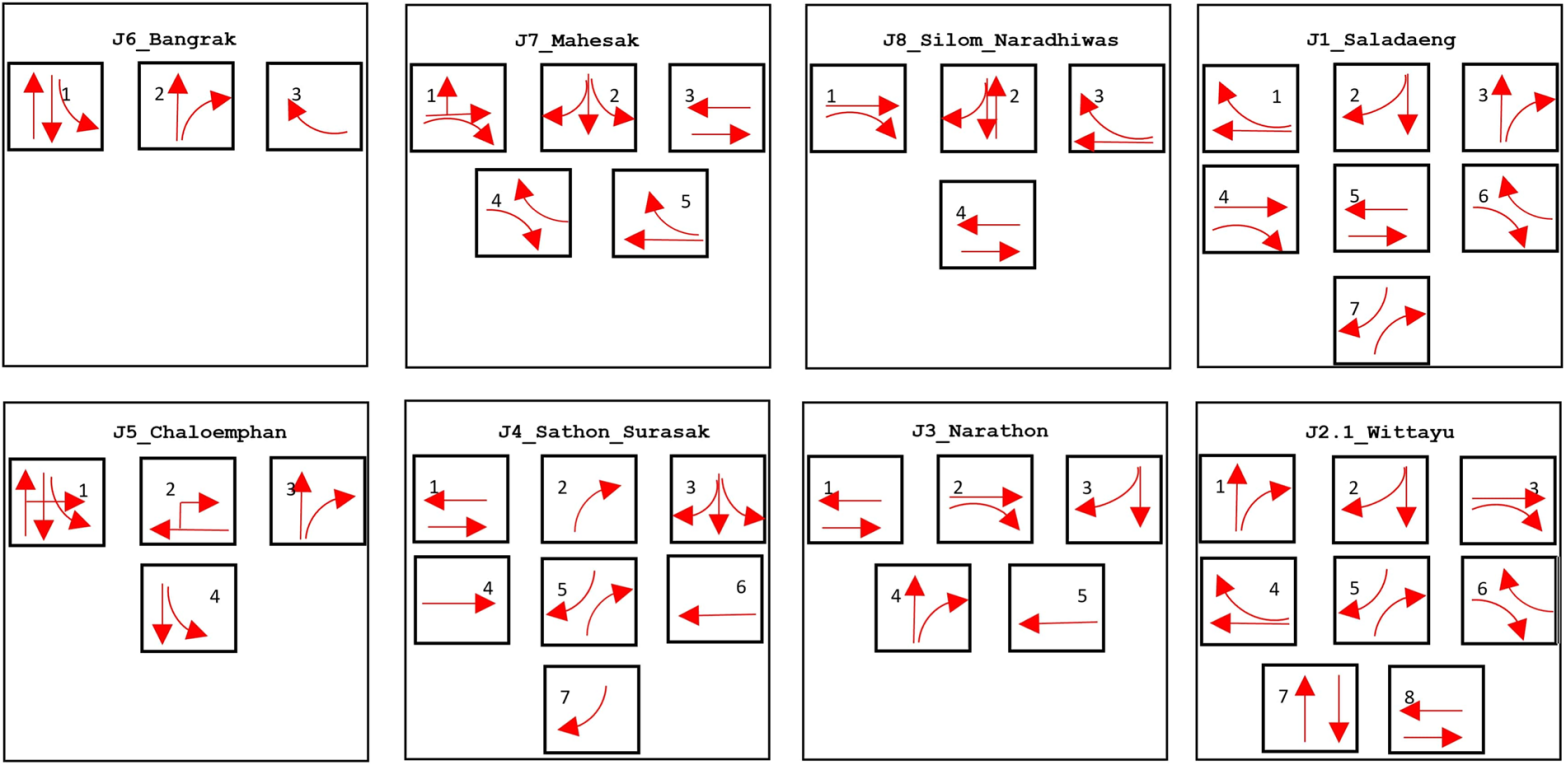
Traffic light phases of each intersection in whole network-wide area on manual traffic data collection day.

**Fig. 10:**
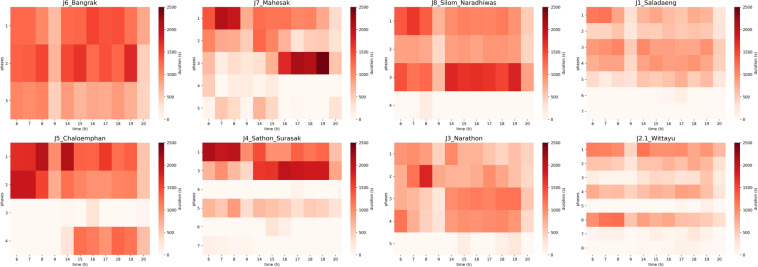
Hourly total duration of each phase used in each intersection in whole network-wide area on manual traffic data collection day.

Compared with the traffic light phases of each intersection as shown in Fig. 9, the total duration of phases (1, 2) in Chaloemphan intersection and phases (1, 3, 5) in Sathon-Surasak intersection are 1000 s to 2500 s per hour, as shown in Fig. 10. The reason is to release the long queue from the west Taksin bridge for morning and evening rush hours, and traffic police have used these phases frequently to control the spillovers effect. Also, traffic police have used this strategy for phases (1, 2, 3) used in Bangrak, Mahesak and Silom-Naradhiwas to prevent queue spillback and release incoming traffic from the off-ramp and ongoing traffic to the on-ramp Sri Rat expressway in the morning and evening rush hours.

According to the observed traffic conditions from the manual collection as shown in Figs. 7, 8 and 10, this clear evidence has recommended to select the Sathorn-Surasak intersection as the critical for inbound and outbound of the business district area, and we have tried automatic data collection using real-world sensors.

### Traffic volume from loop coil sensor and thermal CCTV camera

**Fig. 11:**
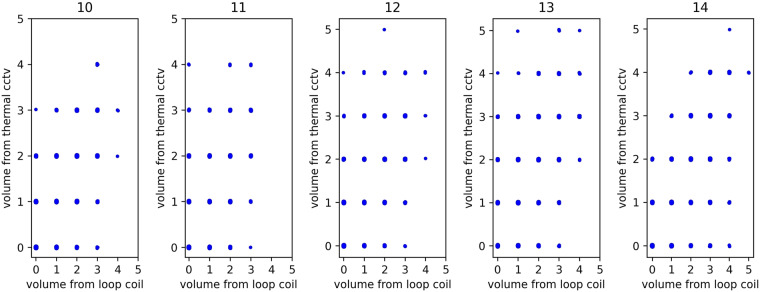
Volume monitored by the loop coil sensors and thermal CCTV cameras on Link 1 every 5 s on June 2, 2016.

Figure 11 shows the traffic volume from loop coil sensors and thermal CCTV cameras on Link 1. Figure 11 shows observed traffic conditions from two types of sensors on Link 1 at the Sathorn-Surasak intersection on example data collected on June 2, 2016. The scattering points in Fig. 11 have been internationally perturbed by small random noise so that one can see the scattering point’s occurrence frequency. The traffic volumes from the loop coil and thermal cameras are positively correlated, as shown in Fig. 11, with captured numbers of vehicles of 0 to 5 for both sensors. The Pearson correlation coefficient^35^ has been used to show the correlation degree between the two types of data. The correlation coefficient is 0.8, which is sufficiently close to 1 and indicates a strong correlation^36^, confirming the validity of using thermal CCTV camera-based sensors to acquire traffic volume. Thus, after confirming the feasibility of using thermal CCTV camera-based sensors, subsequent data collection expansion has been planned by continued use of CCTV camera-based sensors for traffic volume collection over the long term. Note that the software for video signal processing of CCTV and thermal CCTV sensors is the same. The normal grade of CCTV (without thermal detection) has been selected, but occupancy has not been determined during this scaled-up installation because of cost.

**Fig. 12:**
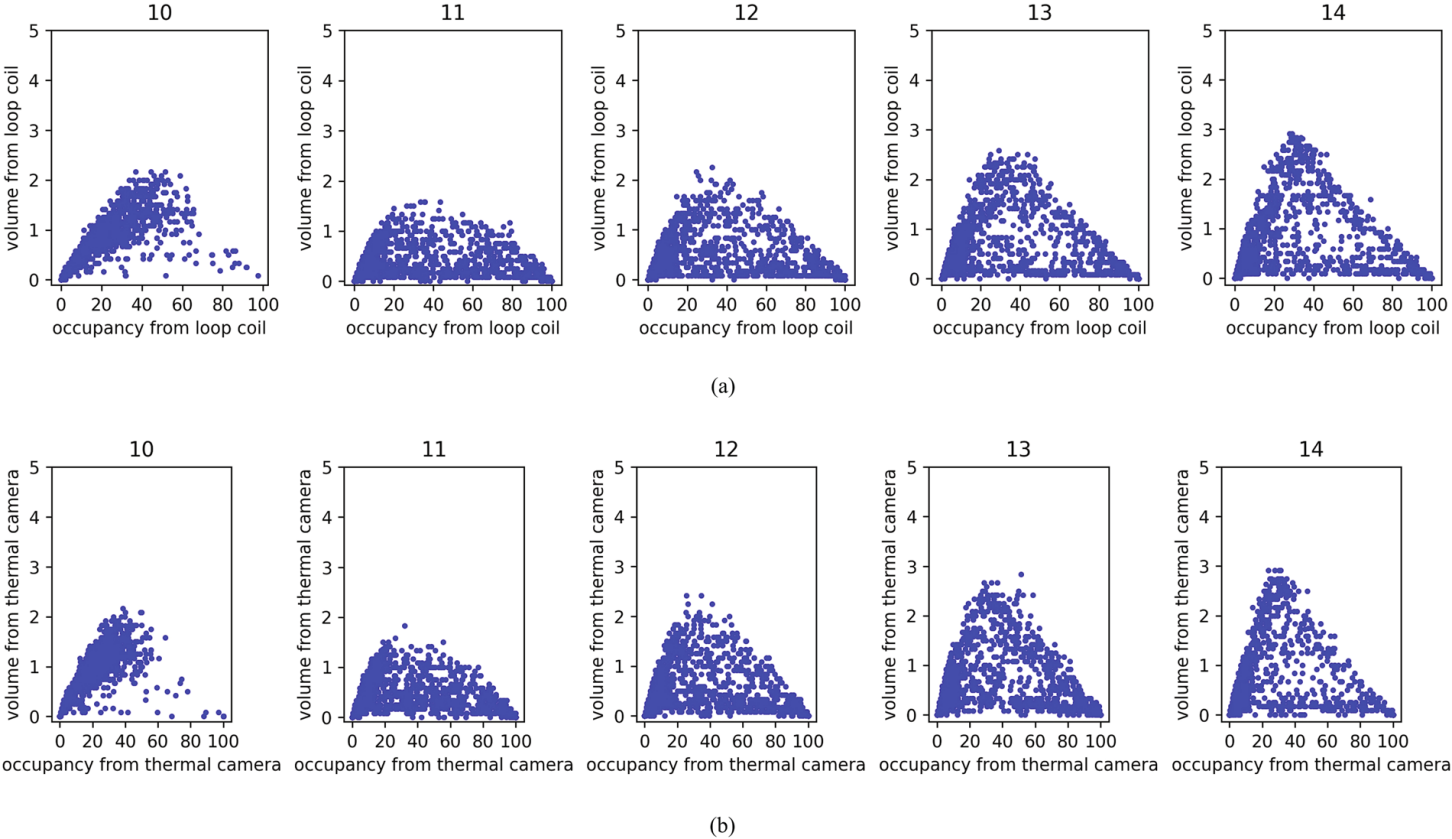
Per-lane fundamental diagram for interrupted flow at critical signalized intersection with the relationship of average per-minute volume and occupancy percentage from each sensor on every lane of Link 1 computed data obtained from (**a**) a loop coil sensor and (**b**) a thermal camera on June 2, 2016.

The fundamental diagram is one of the most basic concepts in traffic flow theory and contains practical information on traffic features. Figure 12(a,b) show typical fundamental diagrams for the outgoing link (Link 1) of the Sathorn-Surasak intersection, which comprises two diagrams with all loop coil and thermal sensors on Link 1 by capturing traffic volume and occupancy relationships. The results show a clear relation between volume and occupancy under real traffic conditions for the outbound critical intersection. Figure 12 shows the tendency of the free-flow region and jam or congestion region in the fundamental diagram. The free flow region corresponds to steady traffic flow at high speeds (and low densities), while the congestion region is characterized by low flow and high densities.

### Traffic volume from CCTV camera

**Fig. 13:**
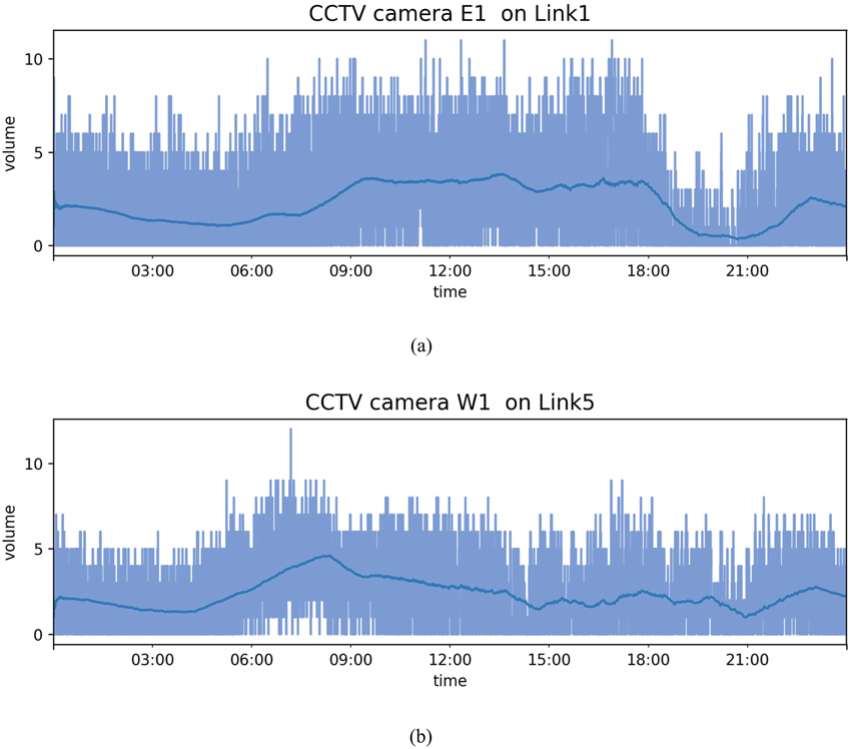
Volume in all measured lanes monitored by CCTV cameras at locations (**a**) E1 on Link 1 and (**b**) W1 on Link 5 collected every 5 s on October 2, 2016.

**Fig. 14:**
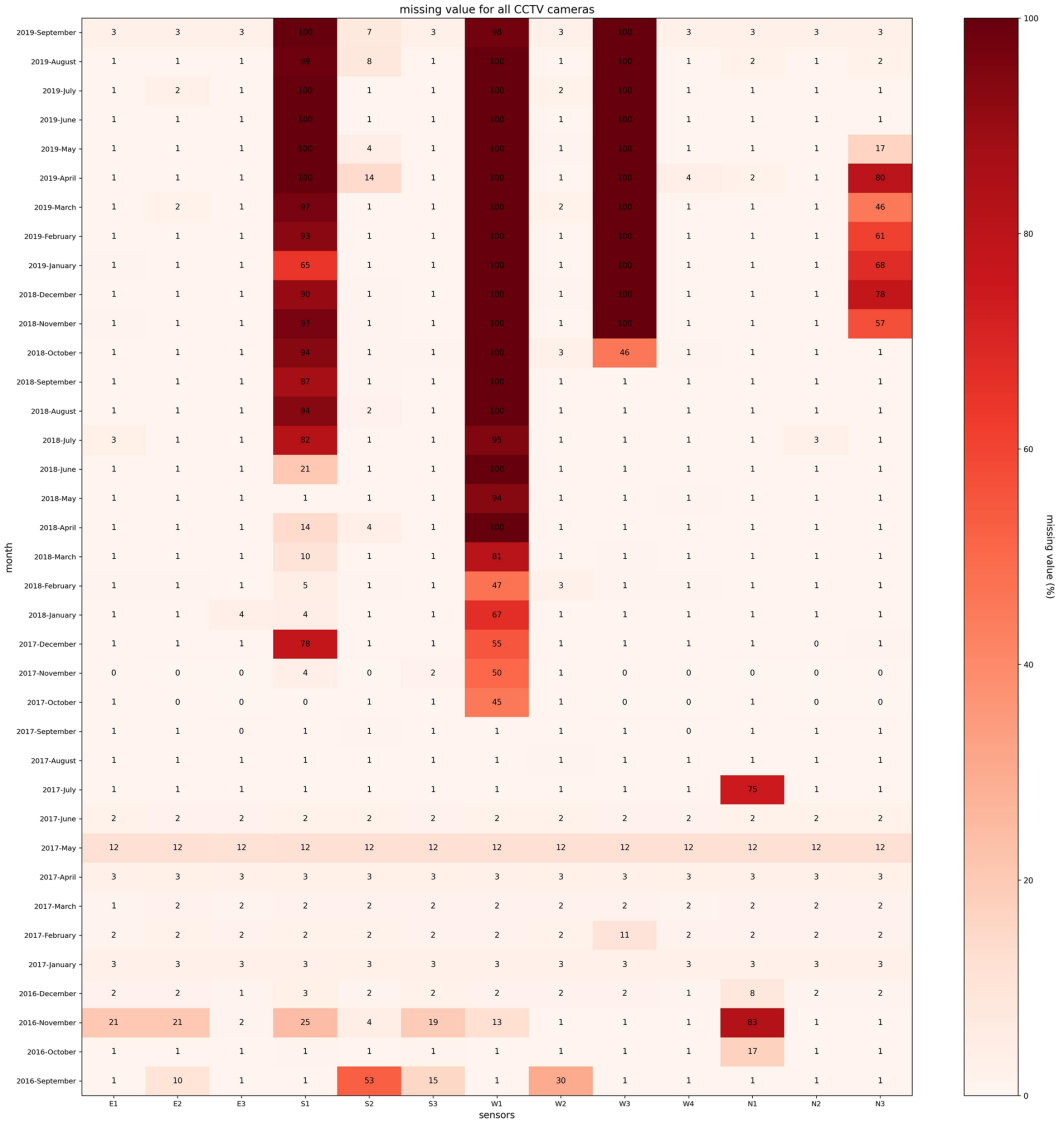
Missing percentage of traffic volume captured by all CCTV cameras on all approaching links of the Sathorn-Surasak intersection.

**Fig. 15:**
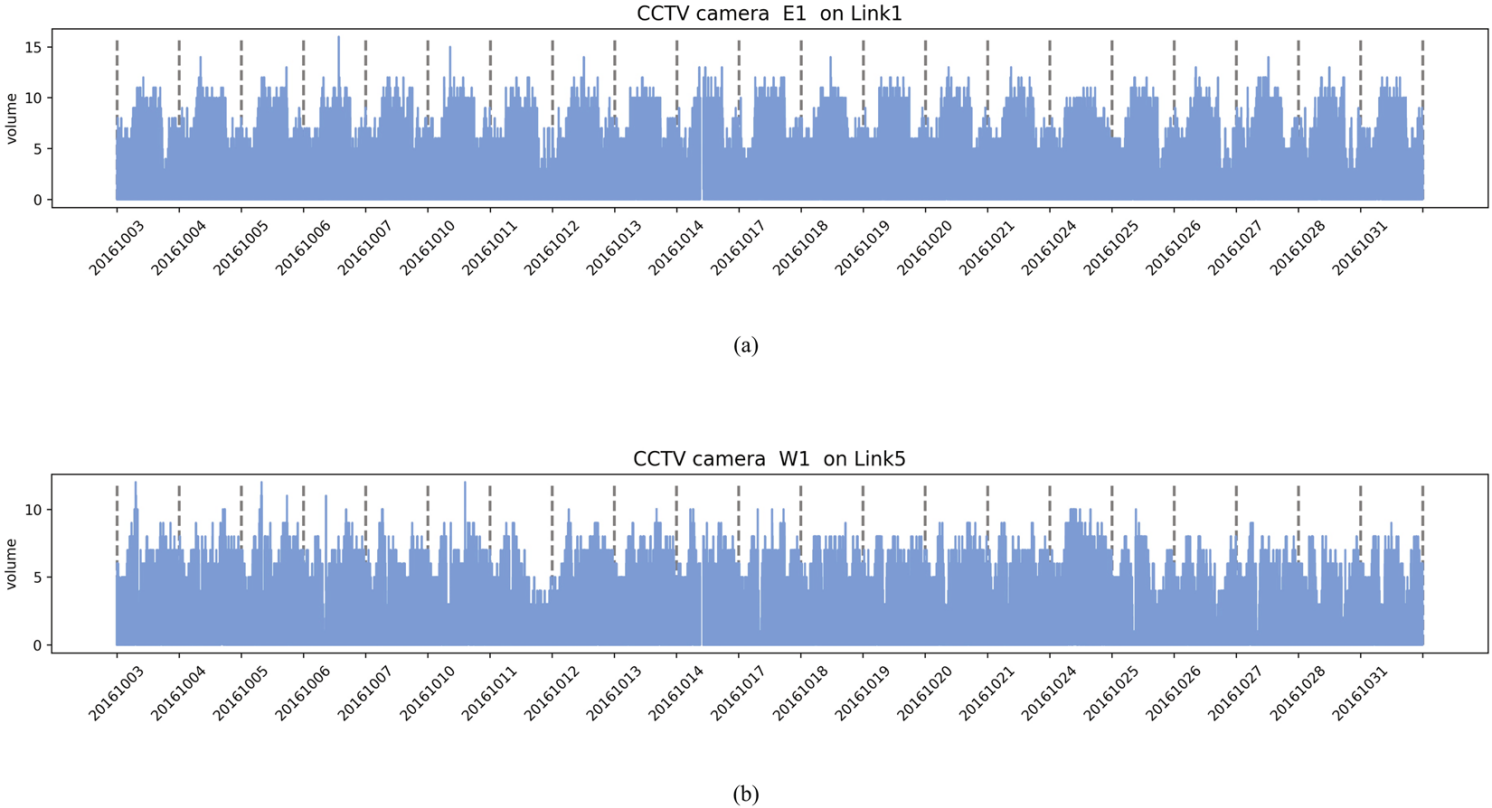
Volume in all the measured lanes monitored by CCTV cameras at locations (**a**) E1 on Link 1 and (**b**) W1 on Link 5 collected every 5 s on a weekday in October 2016.

Traffic volume is a measure used primarily in transportation planning, and daily traffic volume from CCTV cameras indicates how busy the road is. For example, Fig. 13(a,b) show the traffic volume per day every 5 s and moving average volume per hour captured by the CCTV camera at locations E1 on Link 1 and W1 on Link 5 of the Sathorn-Surasak intersection. The missing data plot provides insights into data availability. Missing-value analysis helps address several concerns caused by incomplete data. Figure 14 shows the missing percentage of traffic volume from CCTV sensors on all links (Link 1, Link 3, Link 5, and Link 6) for 37 months. Figure 15(a,b) show the traffic volume pattern captured by the CCTV camera at locations E1 on Link 1 and W1 on Link 5, with a weekday pattern for all of October 2016.

### Hourly total traffic volume from CCTV camera and loop coil sensor

**Fig. 16:**
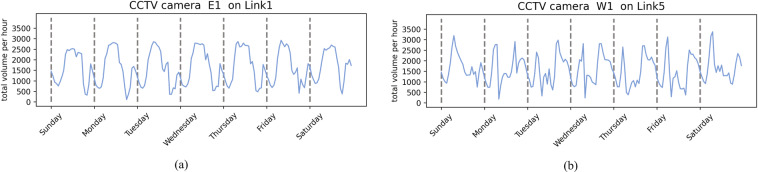
Total volume per hour in all measured lanes monitored by CCTV cameras at locations (**a**) E1 on Link 1 and (**b**) W1 on Link 5 from October 2 to 8, 2017.

The hourly traffic volume from CCTV cameras and loop coil sensors also provide an aggregated measure of the road’s hourly total volume. For example, Fig. 16(a,b) show the hourly traffic volume pattern obtained by the CCTV camera at locations E1 on Link 1 and W1 on Link 5.

**Fig. 17:**
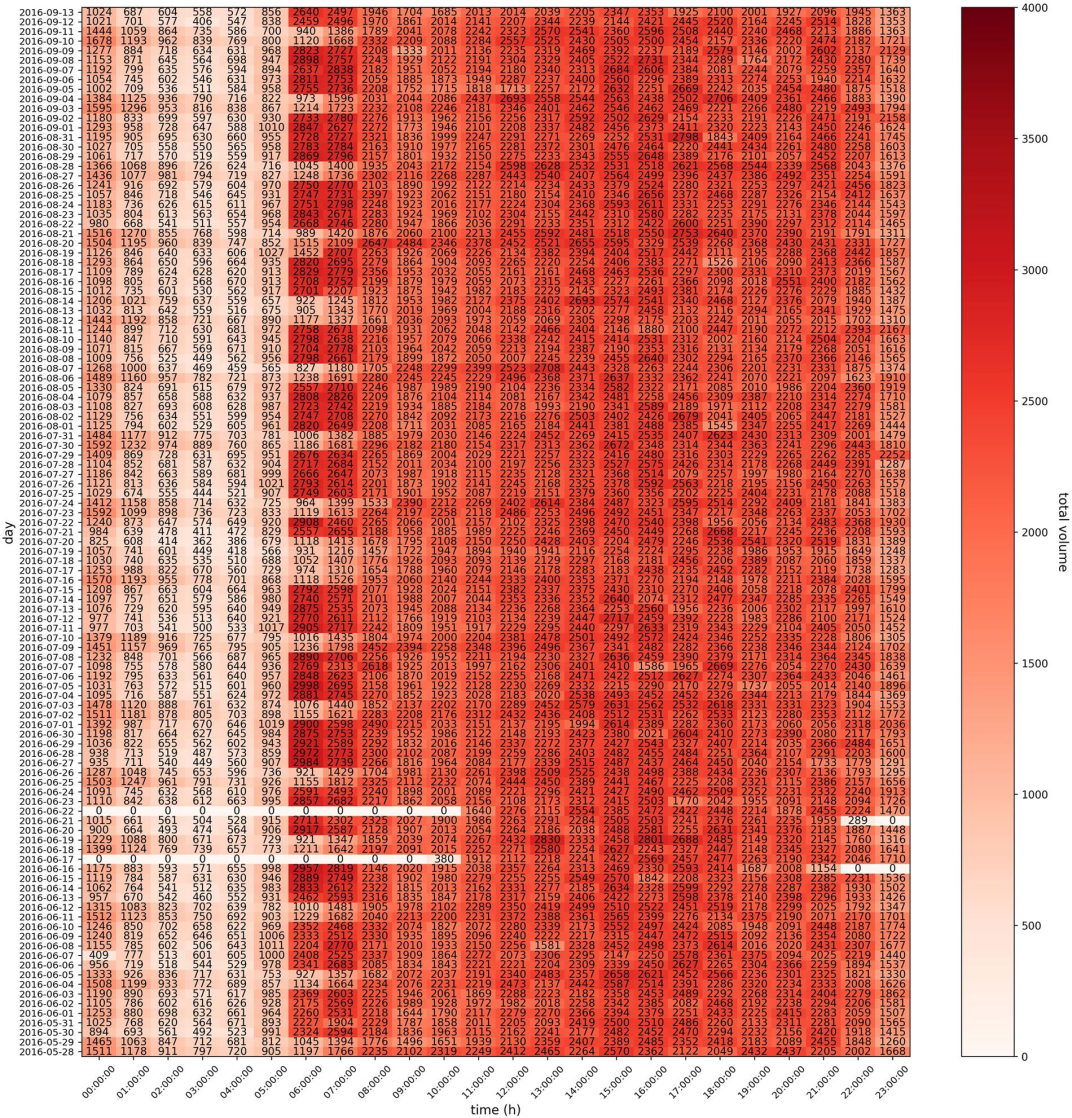
Total volume per hour monitored by loop coil sensors (10, 11, 12, 13, 14) on Link 1 from May 28 to September 13, 2016.

Figure 17 shows the hourly traffic volume obtained by loop coil sensors on Link 1. The total volume is the sum of the volume monitored by each loop coil sensor (10, 11, 12, 13, 14 and 15). The white color with 0 indicates that the sensors are not working at that time. Figure 17 shows the total data availability of loop coil sensors on Link 1 for 110 days, and Fig. 17 shows the periodic pattern of weekend traffic. These data also show the increase in traffic during morning rush hour periods as early as 6 AM, with hourly traffic exceeding 2000 cars; this level of demand persists throughout the entire day until as late as just before 11 PM. This observation emphasizes the traffic density of the critical Sathorn-Surasak intersection.

### Daily total traffic volume monitored by CCTV cameras

**Fig. 18:**
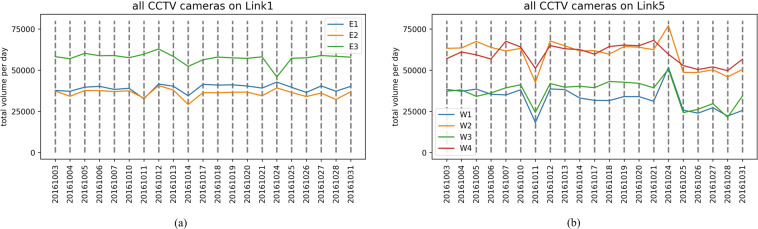
Total volume per day monitored by CCTV cameras on weekdays on (**a**) Link 1 and (**b**) Link 5 in October 2016.

**Fig. 19:**
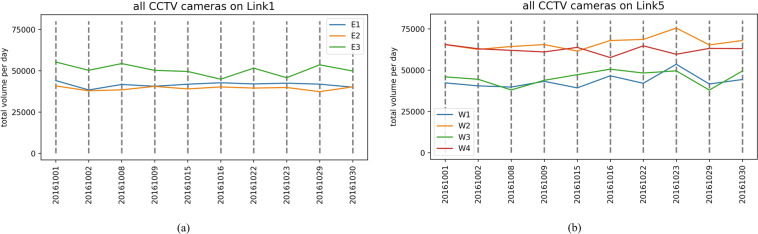
Total volume per day monitored by CCTV cameras on weekends on (**a**) Link 1 and (**b**) Link 5 in October 2016.

The daily total traffic volume monitored CCTV cameras also provides an aggregated measure of the road’s daily total volume. The weekday and weekend daily total traffic volume patterns highlight how busy the road is and how many commuters have used roads that pass through the critical Sathorn-Surasak intersection. For example, Fig. 18(a,b) show the daily total traffic volume monitored by all CCTV cameras on Link 1 and Link 5 on weekdays in October 2016. According to the measured number of vehicles, the reported traffic volume monitored by CCTV cameras located at the intersection is higher than that monitored by other CCTV cameras; for example, E3 on Link 1 is near the Naradhiwat intersection, and W4 on Link 5 is near the Wongwian Yai intersection. Drivers avoid lane changes at intersections in busy areas, and CCTV cameras are intentionally configured to capture data primarily in the middle lanes. In addition, the reported volume from W2 on the inbound link of Link 5 is on the Taksin bridge. Note that vehicle queues occur on this link starting from the Surasak intersection until the Taksin bridge. Similarly, Fig. 19(a,b) show the daily total traffic volume patterns on weekends in October 2016.

Figures 18 and 19 show that commuters use inbound and outbound links of Sathorn-Surasak on both weekdays and weekends, and there is not much difference in traffic demand between weekdays and weekends.

### Estimated queue length using traffic volume from CCTV camera

Signalized intersections are critical nodes in urban road networks. The capacity and control mechanism significantly impact the level of service (LOS) and the connectivity of urban road networks^37^. Signalized intersections are also frequent locations of traffic congestion^38^. The queue length of each link at the intersection can represent the road traffic state intuitively and is the most common index to identify spillovers.

**Fig. 20:**
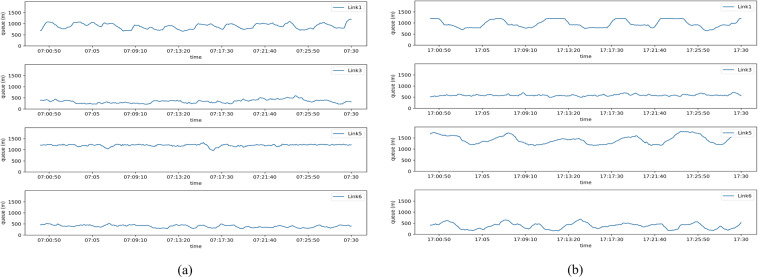
Total queue length of all links approaching the Sathorn-Surasak intersection on October 1, 2016, during (**a**) morning rush hours and (**b**) evening rush hours.

As shown in Fig. 2, CCTV sensors are numbered and located on each link with the opposite direction of traffic movement. The queue length can be estimated by the volume captured by each sensor during the time interval. As shown in Fig. 14, the traffic volume captured by CCTV cameras has missing values. Therefore, the interpolation method has been used to estimate queue length using traffic volume from the CCTV camera. Figure 20(a,b) show the morning and evening queue lengths using interpolated traffic volume from CCTV cameras on all links of the Sathorn-Surasak intersection.

As shown in Fig. 5, the distance between the Sathorn-Surasak intersection and the Wongwian Yai intersection is 3046 m along North Sathorn road, Link 5. Additionally, the distance between the Sathorn-Surasak intersection and the Wittayu intersection is 2760 m along South Sathorn road, Link 1. According to the reported results of queue length in Fig. 20(a,b), the total queue length has reached half of the lengths of Link 1 and Link 5 in both the morning and evening. Moreover, the morning queue length has remained steady without much fluctuation in Link 5 (inbound), and the cycle of queue length was more frequent on Link 1 (outbound) in the morning concerning traffic signal control, as shown in Fig. 20(a). Conversely, the total evening queue pattern is based on the traffic signal tuning of traffic police, as shown in Fig. 20(b). The reported queue length shows that traffic police at the Sathorn-Surasak intersection allow commuters to enter the residential area and exit to the work zone and vice visa using inbound and outbound links.

### Taxi speed

**Fig. 21:**
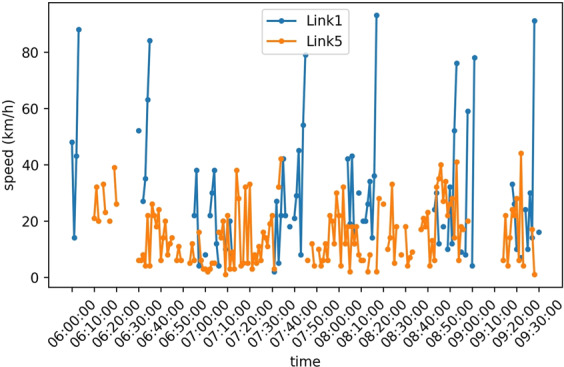
Speed on Link 1 and Link 5 from 6 AM to 9:30 AM on June 2, 2016.

Figure 21 shows the speed of taxis that have been hired to run on target routes during the social experiment of the Sathorn Model project. Figure 21 shows taxi trajectory data on Links 1 and 5 from 6 AM to 9:30 AM. Drivers have tried to commute as typical commuters coming from the residential zone using Link 5 (inbound) and Link 1 (outbound). This trajectory describes how drivers arrived at and left from the Sathorn-Surasak intersection during the morning rush hour.

**Fig. 22:**
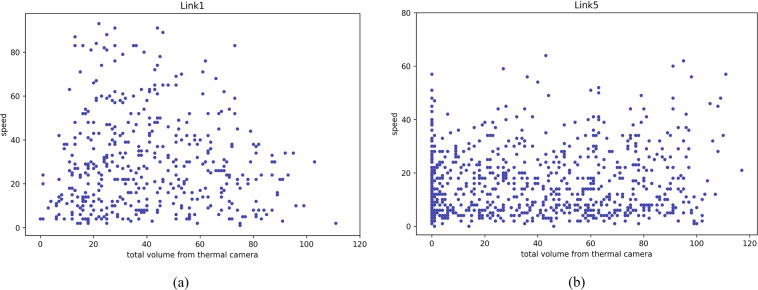
Total volume per minute monitored by thermal cameras on (**a**) Link 1 and (**b**) Link 5 and taxi speed from 6 AM to 9:30 AM for all days on which GPS speed data were collected.

**Fig. 23:**
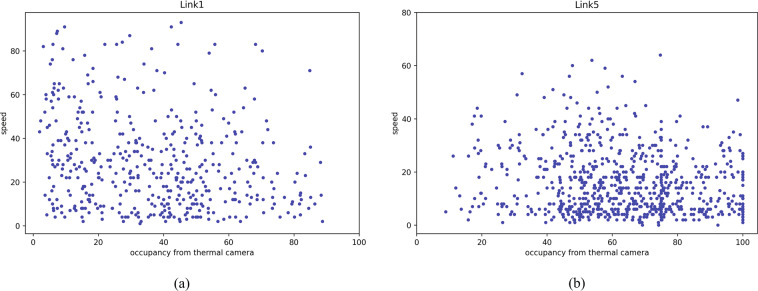
Average occupancy per minute monitored by thermal cameras on (**a**) Link 1 and (**b**) Link 5 and taxi speed from 6 AM to 9:30 AM for all days on which GPS speed data were collected.

The fundamental diagrams of Figs. 22 and 23 show the speed-volume and speed-occupancy relationships for inbound and outbound links of urban signalized intersections using volume and occupancy data collected by thermal cameras and GPS speed data. The plots show that traffic speed in the urban business district zone is low: the average speed is approximately 40 km/h based on the traffic demand and occupancy of the inbound/outbound links. Figure 22(a,b) show the total volume every 1 min monitored by thermal cameras on Links 1 and 5 together with taxi speed from 6 AM to 9:30 AM for all experiment’s target evaluation days with GPS speed data. Figure 23(a,b) show the average occupancy every 1 min monitored by thermal cameras on Links 1 and 5 together with taxi speed from 6 AM to 9:30 AM for all experiment days collecting GPS speed data. From 6 AM to 9:30 AM, commuters from the residential zone used Link 5 (inbound) to enter the work zone of Bangkok’s district Sathorn area. Therefore, the trend of speed-volume is dense in Link 5, and according to the stop-and-go pattern on Link 5 from 6 AM to 9:30 AM, the speed-occupancy trend is also dense, with slower speeds than Link 1 (outbound).

### Signal data

**Fig. 24:**
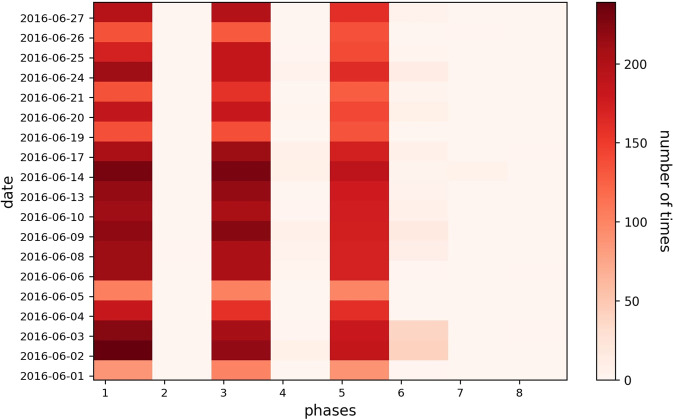
Total number of counts for each phase for the Sathorn-Surasak intersection in June 2016.

There are eight phases used in the Sathorn-Surasak intersection in Fig. 4. Figure 24 shows the total number of counts for each phase used in the Sathorn-Surasak intersection during June 2016. According to the resulting count for each phase, the phases that are primarily used to tune traffic signal lights at the critical Sathorn-Surasak intersection should be identified.

**Fig. 25:**
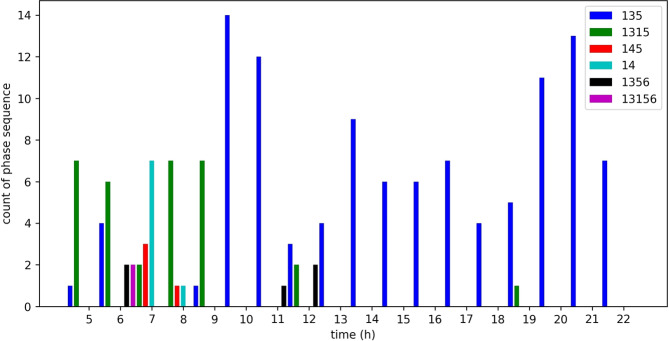
Occurrences of phase sequences per hour on June 2, 2016.

Figure 25 shows the occurrences of regular sequences of phases. This sequence of phases is primarily used in the Sathorn-Surasak intersection. At the critical Sathorn-Surasak intersection, queue spillback occurs when a queue from the downstream intersection occupies all the space on the link and prevents vehicles from the upstream link when the light is green. The phase sequences that are primarily used are [135] and [1315]. The phase sequence [135] represents a periodic sequence of phases 1, 3 and 5, and enables all traffic movement for all links (Link 1, Link 3, Link 5, and Link 6) of the Sathorn-Surasak intersection. However, traffic police have tuned using the phase sequence [1315] to enable Link 5 (inbound) and Link 1 (outbound) more frequently in the morning by using phase 1 twice in the periodic sequence. Between 6 AM and 8 AM, traffic police have tried to tune traffic signal lights based on the particular condition when some spillback occurred.

Between 11 AM and 12 PM (lunchtime), traffic police have also used phase sequences [135], [1315], and [1356] to enable commuters to use both North Sathorn and South Sathorn roads leaving or entering alongside roads. In the afternoon, traffic police have used phase sequence [135], and phase sequence [1315] have been used in some evenings. These results show that all phases have been used with particular traffic conditions at the Sathorn-Surasak intersection. Conversely, some phases, such as phases 2, 7, and 8, have been partially used to enable traffic movements.

## Data Availability

The code implementation was performed in Python using a Jupyter notebook. The Python scripts to perform data preprocessing, visualization and technical validation are available at the sathorndata GitHub repository. (https://github.com/EEM0N/sathorndata.github.io/blob/main/sathorndata.ipynb.)
